# Temporal and Environmental Influences on the Survival of *Escherichia coli* in Poultry Litter‐Based Soil Amendments in Georgia Sweet Onion Production Systems

**DOI:** 10.1111/risa.70330

**Published:** 2026-09-30

**Authors:** Harsimran Kaur Kapoor, Amelia Payne, Krishna Prabha, Laurel L. Dunn, Govindaraj Dev Kumar, Chris Tyson, Manan Sharma, Keith R. Schneider, Aditya Kumar Mishra, Alda F. A. Pires, Patrick Baur, Abhinav Mishra

**Affiliations:** ^1^ Department of Food Science and Technology University of Georgia Athens Georgia USA; ^2^ Center for Food Safety University of Georgia Griffin Georgia USA; ^3^ Vidalia Onion and Vegetable Research Center University of Georgia Lyons Georgia USA; ^4^ Environmental Microbial and Food Safety Laboratory USDA ARS, Beltsville Agricultural Research Center Beltsville Maryland USA; ^5^ Food Science and Human Nutrition Department University of Florida Gainesville Florida USA; ^6^ Department of Statistics University of Georgia Athens Georgia USA; ^7^ Department of Population Health and Reproduction, School of Veterinary Medicine University of California Davis California USA; ^8^ Sustainable Agriculture and Food Systems Program Department of Fisheries, Animal, and Veterinary Sciences University of Rhode Island Kingston Rhode Island USA

## Abstract

Previous studies have shown that pathogen survival in the biological soil amendments of animal origin (BSAAO) is mediated by meteorological conditions. Hence, a 2‐year field study was conducted in Georgia with four soil amendment treatments (heat‐treated poultry pellets, HTPPs; composted poultry litter, PL; unamended, UN) inoculated with *Escherichia coli* TVS 353 (a surrogate for *Salmonella enterica*) and negative control plots (unamended, non‐inoculated). Additionally, 14‐day‐old sweet onion bulbs were transplanted into plots managed by HTPP (O‐HTPP), harvested, and left to cure for 14 days in the field. A linear‐mixed‐effect model (LME) was fitted to *E. coli* population data enumerated from the soil samples collected at specified intervals over 161 days from three replicate plots amended with different soil amendment treatments during both years of the study. Amendment type (UN, PL, HTPP, O‐HTPP) and weather factors (cumulative rainfall (cmrain_2_), average air temperature (at60_12_), relative humidity (RH_12_), soil temperature (ast_12_) for 2 days before sampling, and wind speed 1 day before sampling (W_1_)) were the predictors for the LME models developed. Significant weather predictors that affected the survival of *E. coli* across soil amendment treatment plots were RH_12_ (+0.35) (Year 1); W_1_ (−0.77), at60_12_ (−1.66), and ast_12_ (+1.57) (Year 2). For plots with and without onions, W_1_ (+0.69) (Year 1); at60_12_ (−0.92), ast_12_ (+1.35), cmrain_2_ (+0.37), and W_1_ (−0.36) (Year 2) were significant. All coefficient values represent the estimated change in log_10_ CFU or Most Probable Number (MPN)/g associated with a one‐unit increase in the respective weather predictor, as estimated by the LME model (*p* ≤ 0.05).

## 1 Introduction

Georgia is among the top poultry‐producing states in the United States (US), with over 5000 poultry farms generating approximately 2 million tons of poultry litter (PL) annually (Dunkley et al. 2011). PL is a mixture of poultry excreta, feathers, spilled feed, and bedding materials used in poultry operations. It is often applied directly to agricultural fields as a soil amendment, providing high amounts of nitrogen (N), phosphorus (P), and potassium (K), along with various secondary nutrients (such as calcium, magnesium, and sulfur), as well as micronutrients (Chen and Jiang 2014; Sharma and Reynnells 2016; Tewolde et al. 2016). PL can be pelletized (HTPP) or composted (PL), and it is classified as a biological soil amendment of animal origin (BSAAO) by the Food and Drug Administration (FDA). PL undergoes controlled composting, whereas HTPP is produced by pelleting PL and then thermally processing it at high temperatures (U.S. Government Publishing Office^2024^). Applying BSAAOs plays a crucial role in improving soil structure, enhancing soil texture, increasing water‐holding capacity, and boosting crop yield (Mikha et al. 2014; Rees et al. 2014; Sharma and Reynnells 2016; Gurtler et al. 2018). Its application becomes even more important for farms certified under the US Department of Agriculture (USDA) National Organic Program (NOP). Organic agriculture restricts the use of synthetic inputs, such as fertilizers, pesticides, growth hormones, antibiotics, or sewage sludge (NOP 2011). However, untreated BSAAOs are a potential source of pathogenic microorganisms, including *Escherichia coli* O157:H7, *Listeria monocytogenes*, *Salmonella enterica, Cryptosporidium parvum*, and *Campylobacter* spp. (Bardsley et al. 2021; Litt et al. 2021; Limoges et al. 2022).

Biological soil amendments can pose a threat to public health if they contain human pathogens that contaminate fresh produce during production. The USDA NOP guidelines recommend a 120‐day interval between applying untreated BSAAOs and harvesting crops in contact with the soil, and a 90‐day interval for crops not in contact with the soil (NOP 2011). Due to recent outbreaks of foodborne illness linked to fresh produce, manure‐amended soils are being increasingly evaluated in relation to the survival of enteric pathogens (Carstens et al. 2019; CDC 2020, 2021; McCormic et al. 2022). Recently, the CDC reported a multi‐state outbreak linked to yellow onions contaminated with *E. coli* O157:H7; the investigation reported 104 illnesses, 34 hospitalizations, and one death (CDC 2024). Onions have also been linked to multiple *Salmonella* outbreaks. In 2020, a *Salmonella* outbreak affecting 48 states resulted in 1127 infections and 167 hospitalizations. The outbreak was traced to red onions contaminated with *Salmonella* Newport, resulting in over 1600 reported illnesses across the US and Canada (CDC 2020). In 2021, the CDC reported another multistate outbreak associated with onions, resulting in 1040 illnesses and 260 hospitalizations due to *Salmonella* Oranienburg (CDC 2021). These outbreaks have raised concerns for retail establishments, restaurants, and consumers due to the use of onions as raw ingredients in prepared foods, including salsa, cheese dip, and salad kits.

Understanding the impact of environmental factors and manure type on enteric pathogen levels in amended soil is essential for assessing and managing the food safety risk associated with fresh produce grown in manure‐amended soils. Published studies have shown that the population of foodborne pathogens is affected by the amendment type, temporal factors, and weather factors such as soil micro‐environment (soil moisture, temperature) and environmental effects (rainfall, wind speed, air temperature, and relative humidity) (Habteselassie et al. 2008; Pang et al. 2017, 2018, 2020; Tran et al. 2020; Kapoor et al. 2026). Limoges et al. (2022) found that *E. coli* survived better in soils amended with composted or heat‐treated PL compared to unamended plots in a study conducted in northeastern US soils. Similarly, Shah et al. (2019) reported that *S. enterica* survived longer in soils amended with HTPPs. The thermal processing of HTPP not only reduces pathogen load but also concentrates key nutrients, including organic nitrogen and phosphorus, and lowers moisture content. These changes may influence microbial competition and resource availability, potentially supporting longer *E. coli* survival compared to composted litter or unamended soils. The presence of additional nutrients and organic matter in amended soils is thought to provide a favorable microenvironment for pathogen survival (Nicholson et al. 2005), which underscores the importance of evaluating *E. coli* survival, using these poultry‐litter‐based amendments in Georgia production systems. Sharma and Reynnells (2018) conducted a longitudinal study to determine the survival of *E. coli* in agricultural fields containing different types of BSAAOs at three different sites in the Mid‐Atlantic region of the US. The study reported weather factors (average daily air temperature, average daily rainfall, and initial soil moisture content) accounted for 10% of the variability associated with survival of up to 100 days, and the % variance increased by adding more variables related to the site, year, season, and amendment to the model. Litt et al. (2021) identified rainfall amounts and patterns affecting the survival duration of *E. coli*, along with other predictors related to season, year, and amendment. Quantifying *E. coli* levels in soil provides an important indicator of preharvest contamination risk. However, pathogens present in soil can contaminate produce through multiple routes, including splash from rainfall or irrigation water and direct contact between the edible portion of the crop and the soil. Therefore, this study also evaluated *E. coli* levels on onions before and after curing.

Currently, the FDA has “no objection” to growers following the USDA NOP guidelines or harvesting fresh produce immediately after applying biological soil amendments that have undergone scientifically valid, controlled biological composting processes (FDA 2015). However, the FDA seeks more data to better understand the food safety risks associated with BSAAOs. The quantitative effects of biological soil amendment and of different environmental factors remain undescribed in the literature. Therefore, the objectives of this longitudinal field study in Georgia are (i) to compare the effect of soils amended with composted PL and HTPP on the survival of *E. coli*, (ii) to assess the presence of sweet onions growing on the survival of *E*. coli, (iii) to model the influence of several environmental factors (rainfall, wind speed, air temperature, soil temperature, and relative humidity) on the survival of *E. coli* in soil amended with BSAAOs, and (iv) to determine the effect of curing on the prevalence of *E. coli* in onions.

## 2 Materials and Methods

### 2.1 Location, Field Plot Layout, and Biological Soil Amendment Treatments

This study was conducted twice, in Year 1 (November 1, 2021, to March 21, 2022) and Year 2 (November 7, 2022, to March 28, 2023), at the University of Georgia Vidalia Onion and Vegetable Research Center in Lyons, GA, USA. Each year, the experiment was conducted in production fields measuring 15.2 × 45.7 m^2^, consisting of four biological soil amendments and three replicates each, for a total of 12 randomized treatment plots (Figure 1). The soil treatments were as follows: (i) negative control (C)—unamended and non‐inoculated, (ii) positive control (UN)—unamended and inoculated with *Escherichia coli*, (iii) PL—amended with composted PL and inoculated with *E. coli*, and (iv) HTPP—amended with pelleted PL subjected to thermal processing and inoculated with *E. coli*. Both amendments (composted PL and HTPP [Everlizer, commercially available OMRI‐listed product]) were sourced as finished commercial products. The HTPP product was processed in accordance with OMRI/NOP standards for biological soil amendments of animal origin (BSAAO). These treatment plots were prepared in a completely randomized design, composed of raised beds (1 m × 3 m) with a 2.6 m × 4.6 m buffer gap. The amendments were analyzed for their physicochemical properties by Analytical Services Laboratories (ANSERV) Labs, UF, Gainesville, FL, in Year 1, and by Agricultural and Environmental Services Laboratories (AESL), UGA, Athens, GA, in Year 2 (Tables S1 and S2). The soil at the experimental site is classified as a loamy sand, with a particle‐size distribution of 81.6% sand, 9.0% clay, and 9.4% silt (Kharel et al. 2025). Six plots were transplanted with the Fast Track onion seedlings (Vilmorin Mikado USA, Salinas, CA) to determine the fate of *E. coli*. Of the six onion plots, three were inoculated with *E. coli* and managed with the application of HTPP (O‐HTPP), and the remaining three served as a negative control (O–C) (Figure 1). We followed the cultivation practices outlined in the onion production guide, published by the University of Georgia Cooperative Extension (UGA Extension 2026). Irrigation water from groundwater sources was used, and plots were irrigated with five sprinklers at a rate of 1.79 L/m^2^ for 1 h daily. Pesticides and synthetic fertilizers were routinely applied, totaling 1457 kg/ha for the entire growing season. Although plots were amended with poultry‐litter‐based amendments, the use of synthetic fertilizers reflects the standard soil fertility program at the Research Center for commercial production of sweet onions (UGA Extension 2026).

**FIGURE 1:**
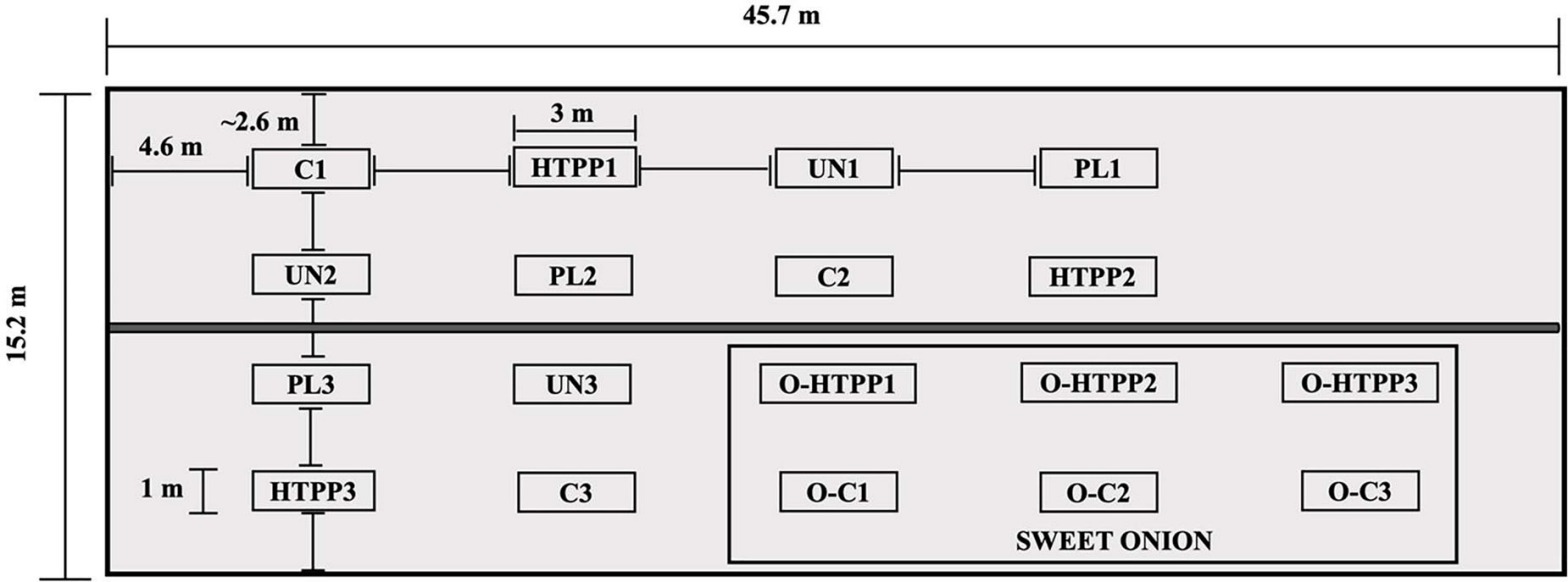
Field plot layout in 2021 and 2022 (Year 1; Year 2) at Lyons, GA, to examine the survival of *Escherichia coli* TVS 353 in soils containing different treatments. Soil treatment plots (1 m by 3 m rectangles) were (i) composted poultry litter with *E. coli* (PL), (ii) heat‐treated poultry pellets with *E. coli* (HTPP), (iii) negative control (C) (unamended and non‐inoculated), and (iv) positive control (UN) (unamended and inoculated with *E. coli*). Six HTPP plots were transplanted with the sweet onion seedlings, represented as (i) managed with heat‐treated poultry pellet (O‐HTPP) and (ii) negative control (unamended and non‐inoculated, O–C). Within each row, plots were separated by 4.6 m, and rows were separated from each other by ∼2.6 m. PL, poultry litter.

### 2.2 Bacterial Culture Preparation

This study utilized a rifampicin‐resistant, nonpathogenic *E. coli* strain, TVS 353 (UC Davis), which is commonly employed as a model for pathogenic *E. coli* in field studies (Tomás‐Callejas et al. 2011; Kapoor et al. 2025; Appolon et al. 2026). As previously described by Kharel et al. (2025), the culture was revived from frozen glycerol stocks stored at −80°C by three subsequent transfers into 10 mL tryptic soy broth with rifampicin (80 µg/mL) (TSBR; BD Diagnostics, Sparks, MD, USA). Tryptic soy agar supplemented with 80 ppm of rifampicin (TSAR; BD Diagnostics, Sparks, MD, USA) was streaked from TSBR with an overnight *E. coli* culture. The TSAR plates were then incubated at 37°C for 24 h and stored at 4°C until further use in the experiment. The day before the experiment, a pure colony of *E. coli* from the TSAR plate was transferred onto 25 mL of TSBR and incubated at 37°C for 24 h. On the day of application to treatment plots, bacterial cells were pelleted by centrifugation (Eppendorf, Hamburg, Germany) at 4000 rpm and 20°C for 10 min. The supernatant was removed, washed, and resuspended twice in 50 mL of 0.1% buffered peptone water (BPW; Difco, Sparks, MD). For the field experiment, 100 mL of bacterial suspension was diluted in 900 mL of 0.1% BPW to achieve a final inoculum level of 8 log_10_ CFU/mL for application to the plots.

### 2.3 Survival of *E. coli* in Biological Soil Amendment Treatments

Composted PL and HTPPs were uniformly spread across each treatment plot at the rate of 680 g of amendment per plot. Next, 1 L of *E. coli* TVS 353 culture was sprayed onto the surface of each soil treatment plot (UN, PL, or HTPP) from approximately 10 to 15 cm above the soil surface using a 15 L backpack sprayer (Model 19001, H.D. Hudson Manufacturing Company, Lowell, MI). The inoculum and amendments were tilled into the soil using a telescoping rotary cultivator (1.5″ tines; Rocklin Industry LLC, Sacramento, CA, USA). The tiller was sanitized between plots using 70% ethanol to prevent cross‐contamination. Soil samples were collected on Days 0, 1, 3, 7, 14, 28, 56, 84, 112, and 140 postinoculation. The soil sample was scooped from a depth of approximately 5 cm using a sterile plastic scoop. Five scoops (disposable soup spoons; PLASTICPRO, China) of approximately 20 g subsamples were collected from different random spots within each treatment plot to create one composite soil sample of 100 g from each plot. The soil samples were collected and stored in 24 oz Whirl‐Pak bags (Nasco, Fort Atkinson, WI), transported to the laboratory in a cooler with ice, and analyzed the same day.

As previously described by Kharel et al. (2025), each 30 g soil sample (from the composite soil sample of each plot) in a sterile Whirl‐Pak bag was hand‐massaged for 1 min before being diluted in 120 mL of 0.1% BPW and mixed to enumerate *E. coli* populations. Diluted samples were further serially diluted, and 0.1 mL aliquots were plated on TSAR plates and then incubated at 42°C for 24 h. Bacterial concentrations from plate counts were expressed as log_10_ CFU/g. When colony counts fell below the limit of detection (LOD) of 0.70 log_10_ CFU/g, *E. coli* populations were quantified using the Most Probable Number (MPN) method. For performing MPN, 1 mL of 2× TSBR was added to the first column of a 48‐well MPN block (VWR, Radnor, PA), and each well of the seven subsequent columns was filled with 1.8 mL of 1× TSBR. A 1 mL aliquot was taken from the Whirl‐Pak bag and added to the first column of the MPN block, followed by six‐fold serial dilutions by transferring 200 µL from the first well into the second well, mixing, then transferring 200 µL from the second well into the third, and repeating into each subsequent well. The MPN blocks were then incubated at 42°C for 24 h. The next day, *E. coli* growth was confirmed by channel streaking 5 µL of each dilution onto TSAR plates. The channel‐streaked plates were incubated at 42°C for 24 h. Colonies with morphologies similar to *E. coli* TVS 353 were identified, and MPN values (MPN/g) were calculated using the US Environmental Protection Agency calculator (EPA 2022). Bacterial concentrations from MPN values were expressed as log_10_ MPN/g (McKenzie‐Reynolds et al. 2025). The LOD for MPN counts was −0.24 log_10_ MPN/g.

### 2.4 Survival and Fate of *E. coli* in Sweet Onion Plots Containing HTPPs

Fast‐track onion seedlings (*Allium cepa*) were manually transplanted on Day 14 post‐amendment and inoculation. On Day 147 postinoculation, onions were manually pulled from the soil and placed on the soil surface. Each onion was left in the field after being removed from the ground to cure for 14 days (161 days postinoculation). Onion plots were inoculated in a manner similar to that mentioned above. One liter of bacterial culture was sprayed onto the surface of O‐HTPP plots, and soil samples were collected on similar days as above, from 0 to 140, in addition to 147 and 161 postinoculation, and enumerated in the same manner as previously described.

During both years, a total of 15 onions from five sampling sites in each of three O‐HTPP plots were sampled for enumeration on the day when onions were lifted from the ground. The remaining onions were left in the field for 14 days to cure. After curing, a total of 15 onions were again sampled for enumeration, 161 DPI. For each onion sampled, bulk residual soil was removed, and leaves and roots were cut using sterile scissors; the outer dried skin layers of the onion bulb were retained during enumeration. As previously described by Kharel et al. (2025), onion samples were collected and stored in 24 oz Whirl‐Pak bags, transported to the laboratory in a cooler with ice, and analyzed the same day. Each sample contained one onion, which was combined in a Whirl‐Pak filter bag with 100 mL 1× TSBR. As with the soil samples, MPNs were performed in the same manner when colony counts fell below the LOD of 0.70 log_10_ CFU/g. The LOD for MPN counts was −1.22 log MPN/onion.

### 2.5 Field Environmental Data

Field environmental data was collected from the Georgia Automated Environmental Monitoring Network (georgiaweather.net). Table 1 shows the weather‐related variables used in the model to explain the variability in *E. coli* survival each year. As the soil samples were collected between 8:00 and 9:00 a.m. on each sampling day, the corresponding meteorological variables were calculated as daily averages starting the day before sampling.

**TABLE 1:** Description of weather variables used in the model for Years 1 and 2.

Weather variables	Description (units)
cmrain_2_	Cumulative rainfall 2 days before sampling (mm)
RH_12_	Average daily relative humidity 2 m above the surface 2 days before sampling (%)
at60_12_	Average daily air temperature at a height of 60 cm above ground 2 days before sampling (°C)
ast_12_	Average soil temperature at a depth of 5 cm 2 days before sampling (°C)
W_1_	Average wind speed calculated for a duration of 15 min, 1 day before sampling (km/h)

### 2.6 Statistical Analysis

The *E. coli* population data generated from this longitudinal study were used to fit a linear‐mixed‐effects model (LME) to quantify the impact of different biological soil amendment treatments, with or without onions (UN, PL, HTPP, O‐HTPP), and of environmental factors. Two LME models were developed, accounting for a repeated‐measures design and variability among the replicated treatment plots: One modeled *E. coli* survival in biological soil amendment treatments (UN, PL, HTPP), and another modeled *E. coli* survival in HTPP‐managed plots with and without onions. Both were fitted individually for Years 1 and 2 using the “nlme” package in R version 4.3.1 (Pinheiro et al. 2007). The outcome of the Years 1 and 2 LME models was the log_10_ *E. coli* population, calculated by combining plate counts until colony counts fell below the LOD, then using MPN values thereafter. Previous studies have also combined the colony counts with MPN values after the plate counts reached LOD because CFU and MPN differences are insignificant in changing the interpretation of results (Cowburn et al. 1994; Bardsley et al. 2021; Lee et al. 2021; Murphy et al. 2022).

The term “mixed effect” in the LME denotes a model that incorporates both fixed‐ and random‐effect terms. In this study, the fixed‐effect term was used to model the average trend in the *E. coli* population as enumerated on the sampling days, along with the potential influence of field environmental conditions and soil amendment treatment, with or without onions. Accordingly, in the LME accounting for the survival of *E. coli* in soil treatment plots containing different biological soil amendments, fixed effects for the day, soil amendment treatment (UN, PL, HTPP), and weather variables were included. In the LME model, accounting for the persistence of *E. coli* in HTPP plots with and without onions, fixed effects for the day, HTPP treatment plots (with and without onions, denoted as O‐HTPP and HTPP), and weather variables were incorporated. All continuous fixed‐effect weather predictors were centered and scaled before model fitting, ensuring they were on a comparable scale (Brown 2021). Additionally, the random effect term was used to model the extent to which these trends vary across replications of soil treatment plots within each LME. To account for the autocorrelation of observations within each treatment plot, the correlation structure was specified as corAR1, a first‐order autoregressive correlation. The reason for using corAR1 was that soil samples were collected more frequently immediately after inoculation, followed by wider intervals due to the higher autocorrelation of the *E. coli* population during the initial days compared to later postinoculation intervals.

Furthermore, the weather variables included in the model were assessed for statistical significance, and those with *p* ≤ 0.05 were considered significant contributors to *E. coli* survival. The significant weather predictors were further analyzed using median‐based *z*‐scoring or percentile‐based methods to identify their extremes during the period in each year when this study was conducted in the field (Iglewicz and Hoaglin 1993; Wilks 2011; Leys et al. 2013; IBM Cognos Analytics 2025). The values of conditional *R* ^2^ evaluate each of the model's total explanatory power and its part related to fixed effects by the value of marginal *R* ^2^. Unless stated otherwise, all statistical analyses were performed using R version 4.3.1.

## 3 Results

### 3.1 *Escherichia coli* Survival Trend in Manure‐Amended Plots With and Without Sweet Onions

The survival of *E. coli* was higher in soils amended with HTPP and PL than in unamended plots; however, this difference was significant only in Year 1 (Table 3). During Year 1, the application of HTPP and PL significantly increased the survival of *E. coli* by 1.3 and 1.2 log_10_ CFU or MPN/g compared to the unamended plot (*p* < 0.05) (Table 3). In Year 2, the magnitude of the increase was smaller (0.6 log_10_ CFU or MPN/g for HTPP relative to UN) and not statistically significant (Table 3). Transplanting onions in soils managed with HTPP did not significantly affect the survival of *E. coli* (Table 3). In this study, the population of *E. coli* decreased significantly (*p* < 0.001) by 161 DPI in Years 1 and 2, regardless of the presence or absence of sweet onions (Table 3). Table 2 shows the *E. coli* populations in manure‐amended plots with and without sweet onions at the beginning and end of the study during both years. No rifampicin‐resistant *E. coli* was detected in the negative control plots in both years, indicating no cross‐contamination between the treatment plots throughout the experiment.

**TABLE 2:** *Escherichia coli* populations in agricultural soils amended with poultry litter and heat‐treated poultry pellets with or without sweet onions at beginning and end of the study in Years 1 and 2.

	Year 1		Year 2	
Soil treatment plots	*E. coli* population at the beginning of the study	*E. coli* population at the end of the study	*E. coli* population at the beginning of the study	*E. coli* population at the end of the study
Unamended (UN)	4.35 ± 0.74	−0.24 ± 0.00	5.69 ± 0.14	−0.24 ± 0.00
Poultry litter (PL)	4.89 ± 0.96	1.60 ± 1.83	5.81 ± 0.14	−0.24 ± 0.00
Heat‐treated poultry pellet (HTPP)	4.13 ± 0.77	1.57 ± 0.38	5.67 ± 0.13	0.96 ± 0.24
Onion plots managed by application of HTPP (O‐HTPP)	4.15 ± 0.89	−0.24 ± 0.00	5.63 ± 0.39	−0.24 ± 0.00

*Note*: Values are expressed as mean log_10_ CFU or MPN/g ± standard deviation (*n = 3*). Beginning of the study refers to Day 0; end of the study refers to Day 140 for the soil treatment plots (UN, PL, HTPP), and Day 161 postinoculation for the onion plots managed by HTPP (O‐HTPP). Once the microbial counts reached the detection limit by spread plating (0.70 log_10_ CFU/g), subsequent analysis was carried out to increase the limit of detection to −0.24 log_10_ MPN/g using the Most Probable Number technique.

### 3.2 Temporal Differences of Environmental Factors on the Survival of *E. coli* in Manure‐Amended Soils With and Without Sweet Onions

#### 3.2.1 Effect of the Weather Factors on the Survival of *E. coli* in Soil‐Only Plots Amended With Poultry‐Litter‐Based Amendments

Figure 2 presents the daily averages of each of the weather factors during Years 1 and 2 from 2 days before the start of the field experiment to 161 DPI. During Year 1, a 1‐U increase in the relative humidity averaged over the 2 days before sampling (RH_12_) significantly increased the *E. coli* survival by 0.350 log_10_ CFU or MPN/g (*p* < 0.05) (Table 3). In Year 2, a one‐unit increase in wind speed 1 day prior (W_1_) and air temperature averaged over 2 days before sampling (at60_12_) significantly decreased the *E. coli* survival by 0.769 and 1.661 log_10_ CFU or MPN/g, whereas soil temperature averaged over 2 days before sampling (ast_12_) increased the survival by 1.568 log_10_ CFU or MPN/g (*p *< 0.001) (Table 3).

**FIGURE 2:**
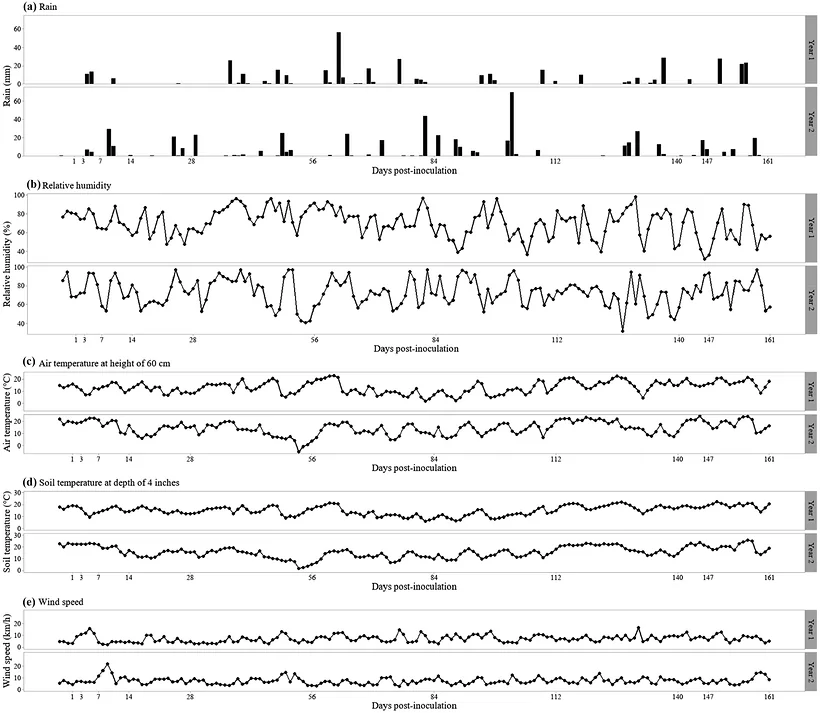
Daily average field environmental factors: (a) rain, (b) relative humidity, (c) air temperature, (d) soil temperature, and (e) wind speed from 2 days before the start of the experiment to Day 161 postinoculation. The *x*‐axis is marked with the days of soil sampling postinoculation (1, 3, 7, 14, 28, 56, 84, 112, 140) and days of onion sampling (Day 147, harvesting, and before curing of onions, Day 161, after curing), and the *y*‐axis represents the corresponding weather parameter.

**TABLE 3:** “Year 1” and “Year 2” results of linear‐mixed‐effect model that were developed to model: (i) survival of *Escherichia coli* in soil treatment plots containing different biological soil amendments (UN, PL, HTPP), and (ii) survival of *E. coli* in soil of HTPP plots with and without onions (O‐HTPP, HTPP).

			Year 1				Year 2			
Model type	Fixed factors		Effect estimate	*p* value	Conditional *R* ^2^	Marginal *R* ^2^	Effect estimate	*p* value	Conditional *R* ^2^	Marginal *R* ^2^
i. Survival of *E. coli* in soil treatment plots	Soil treatment (positive control, UN = reference)^a^	Soil amendment poultry litter (PL)	1.209	0.007^*^	0.62	0.62	−0.087	0.741	0.88	0.88
		Soil amendment heat‐treated poultry pellet (HTPP)	1.353	0.004^*^			0.592	0.058		
	Day of sampling	Days postinoculation	−0.016	0.001^*^			−0.031	<0.001^*^		
	Weather factor variables	cmrain_2_	0.164	0.688			−0.027	0.795		
		RH_12_	0.350	0.044^*^			0.237	0.170		
		at60_12_	−0.706	0.153			−1.661	<0.001^*^		
		ast_12_	0.668	0.233			1.568	<0.001^*^		
		W_1_	0.154	0.365			−0.769	<0.001^*^		
ii. Survival of *E. coli* in the soil of onion plots	Plots managed by application of heat‐treated poultry pellets (HTPP) with and without sweet onions (soil amendment treatment plot containing HTPP = reference)^b^	Onion plot (O‐HTPP)	−0.166	0.563	0.69	0.67	−0.126	0.732	0.85	0.85
	Day of sampling	Days postinoculation	−0.021	<0.001^*^			−0.031	<0.001^*^		
	Weather factor variables	cmrain_2_	0.226	0.532			0.374	0.004^*^		
		RH_12_	−0.041	0.805			−0.289	0.167		
		at60_12_	−0.058	0.876			−0.921	0.040^*^		
		ast_12_	0.056	0.885			1.352	0.001^*^		
		W_1_	0.691	<0.001^*^			−0.361	0.011^*^		

*Note*: “^*^” indicates a significant fixed factor for the enumerated *E. coli* population.

^a^Year 1: Variance component associated with the random effect of three replications of each of the soil treatment plot (Std. Dev. = 7.128921e^−05^). Year 2: Variance component associated with the random effect of three replications of each of the soil treatment plot (Std. Dev. = 3.990912e^−05^).

^b^Year 1: Variance component associated with the random effect of three replications of each of the heat‐treated poultry pellet plots with and without sweet onions (Std. Dev. = 0.207) suggests minimal variability. Year 2: Variance component associated with the random effect of three replications of each of the heat‐treated poultry pellet plots with and without sweet onions (Std. Dev. = 4.252402e^−05^) suggests minimal variability.

Furthermore, the significant weather parameters were compared descriptively to identify the contrasting temporal differences in the distribution and frequency of extreme weather events (Figure 3). Extreme high and low events for each weather variable were identified as median *z*‐score values less than −2 or greater than 2 in Years 1 and 2 (Wilks 2011; IBM Cognos Analytics 2025). High wind speed events (*z* > 2) occurred more frequently and earlier in Year 2 compared to Year 1. The days identified as high extremes throughout the present study ranged from 12 to 21 km/h. These cumulatively increased early in the season, starting at 5 DPI, and clustered at 8–10, 49–52, and 160 DPI during Year 2 (Figure 3a). Low RH events (*z* < −2) were observed in both years of the study; however, their timing and frequency differed. In Year 1, low RH extremes, ranging from 35% to 40%, occurred from Days 53 to 55. In contrast, Year 2 observed low RH extremes in a similar range, clustered during the late season between Days 89 and 139 (Figure 3b). Similarly, low/cold air and soil temperature events (*z* < −2) with temperatures ranging from −5°C to 0.8°C and 2°C to 4°C were observed from 53 to 55 DPI during Year 2. In contrast, Year 1 observed a decline in air and soil temperatures below the seasonal median only at 82 DPI (Figure 3c,d).

**FIGURE 3:**
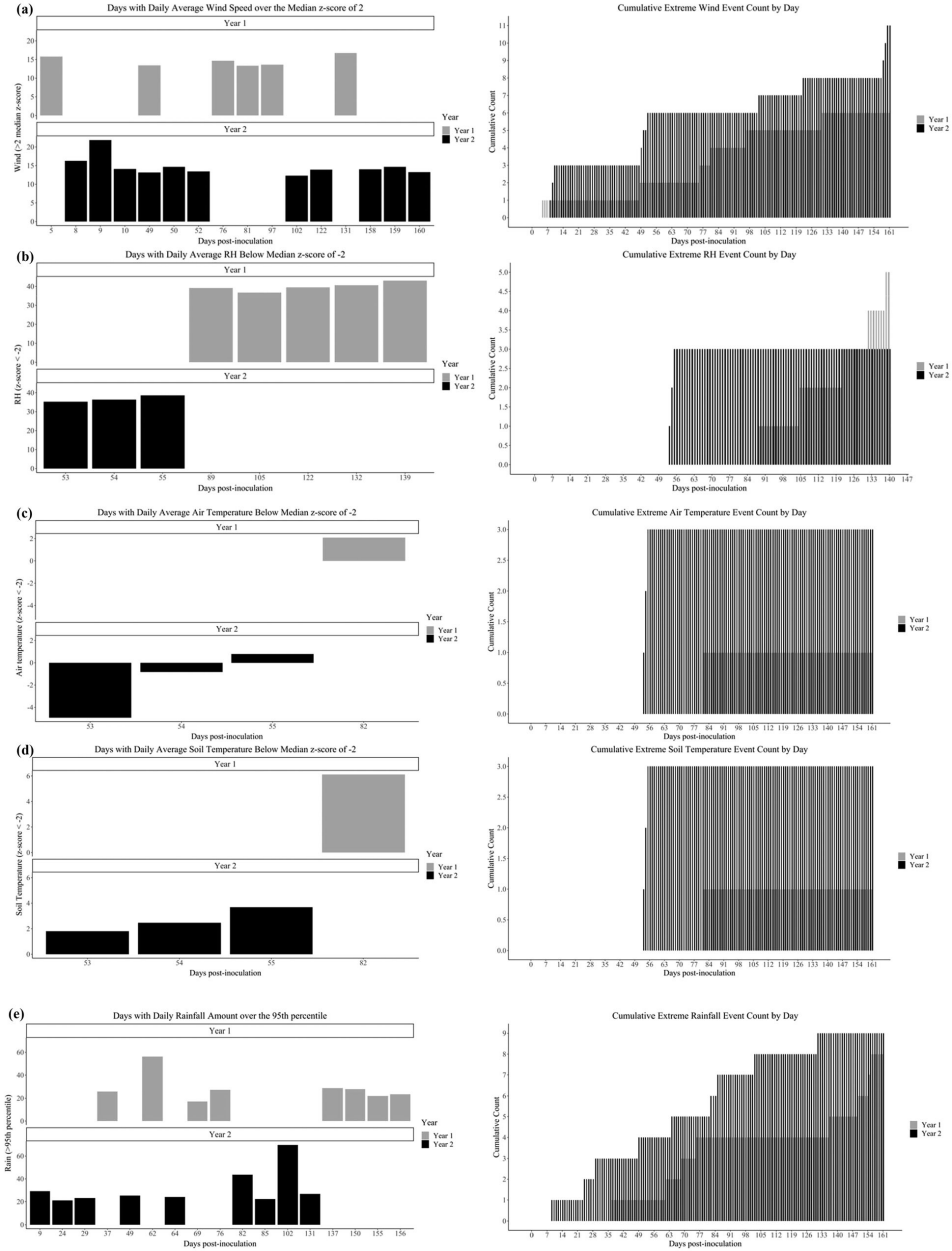
Temporal distribution and cumulative count of extreme weather events in Years 1 and 2. (a) Extreme high events of wind speed with a median *z*‐score of >2, (b) extreme low events of relative humidity (RH) with a median *z*‐score of <−2, (c) extreme low events of air temperature with a median z‐score of <−2, (d) extreme low events of air temperature with a median *z*‐score of <−2, and (e) rainfall extreme events were identified using the 95th‐percentile threshold of daily rainfall totals. Bar plots on the left display the days and values of extreme events, whereas those on the right represent the cumulative distribution of extreme events throughout the study, ranging from 0 to 161 days postinoculation.

#### 3.2.2 Effect of the Weather Factors on the Survival of *E. coli* in HTPP Plots With and Without Sweet Onions

In Year 1, a 1‐U increase in wind speed 1 day prior (W_1_) significantly increased *E. coli* survival by 0.691 log_10_ CFU or MPN/g (*p* < 0.001) (Table 3). During Year 2, a 1‐U increase of W_1_ and air temperature averaged over 2 days before sampling (at60_12_) were associated with significant decreased in the *E. coli* survival by 0.360 and 0.921 log_10_ CFU or MPN/g (*p* < 0.05), whereas cumulative rainfall over 2 days before sampling (cmrain_2_) and soil temperature averaged over 2 days before sampling (ast_12_) increased the survival by 0.374 and 1.352 log_10_ CFU or MPN/g (*p* < 0.01) (Table 3).

As previously mentioned, significant weather parameters were compared descriptively to identify the contrasting temporal differences in extreme weather events using robust median‐*z* scoring. For the extreme rainfall events, a percentile‐based method was employed. Sporadic extreme rainfall events were identified as more than the 95th percentile (Figure 3). Previous environmental studies have also used the 95th‐percentile threshold to assess seasonal trends and selected daily totals equal to or exceeding the 95th percentile as extreme (Łupikasza and Łupikasza 2016; Schär et al. 2016; Zhang et al. 2016). In Year 1, extreme rainfall events occurred primarily during the late season, between 62 and 156 DPI. In contrast, Year 2 exhibited earlier occurrences, with extreme rainfall events recorded as early as 9 DPI and a cluster of events observed between 24 and 105 DPI (Figure 3e). The cumulative count of extreme rainfall days also increased more rapidly in Year 2 compared to Year 1 (Figure 3e).

### 3.3 Fate of *E. coli* in the Sweet Onions Growing in the Field

Across both years, *E. coli* prevalence on onions declined substantially after field curing, but the magnitude of reduction differed between years. In Year 1, prevalence decreased from 66.67% (10/15) to 6.67% (1/15) before and after curing, and the average population of positive onions decreased from 2.51 log_10_ MPN/onion to the LOD (−1.22 log_10_ MPN/onion). Meanwhile, the *E. coli* levels in the soils sampled from O‐HTPP plots decreased from 1.90 log_10_ MPN/g (before curing) to undetectable (after curing) (Table 4a), whereas, in Year 2, prevalence decreased from 60% (9/15) to 40% (6/15). The average population on positive onions decreased from −0.47 log_10_ MPN/onion to −0.097 log_10_ MPN/onion, and *E. coli* levels in the soils sampled from O‐HTPP plots decreased from 1.37 log_10_ MPN/g to undetectable before and after curing (Table 4b).

**TABLE 4:** *Escherichia coli* population and prevalence in heat‐treated poultry pellet‐managed plots with onions (O‐HTPP) before and after curing in Years 1 and 2.

(a) Year 1		
	Average *E. coli* population	% Onion positives (out of 15)
*Before curing (Day 147)*		
Onion (log_10_ MPN/onion)	2.51 ± 2.64	66.67
Soil (log_10_ MPN/g)	2.06 ± 1.91	
*After curing (Day 161)*		
Onion (log_10_ MPN/onion)	−1.24 ± 0.00	6.67
Soil (log_10_ MPN/g)	−0.24 ± 0.00	
(b) Year 2		
*Before curing (Day 147)*		
Onion (log_10_ MPN/onion)	0.59 ± 0.58	60
Soil (log_10_ MPN/g)	1.44 ± 0.23	
*After curing (Day 161)*		
Onion (log_10_ MPN/onion)	1.03 ± 1.20	46.67
Soil (log_10_ MPN/g)	−0.24 ± 0.00	

*Note*: The table shows the average *E. coli* concentration of positive onion samples and average *E. coli* counts from three replicate soil samples collected from O‐HTPP plots (limit of detection, LOD of O‐HTPP and HTPP is −1.22 log_10_ MPN/onion and −0.24 log_10_ MPN/g).

## 4 Discussion

This study applied an LME model to identify significant weather parameters and quantified their influence on the survival of *E. coli*. To fulfill the objectives of the study to understand the impact of temporal and environmental variation, two LME models for each year have been developed in this study: (i) Survival of *E. coli* in soil treatment plots containing different biological soil amendments (UN, PL, HTPP) and (ii) survival of *E. coli* in HTPP‐managed plots with and without onions (O‐HTPP, HTPP). Differences in the frequency and magnitude of extreme environmental variables between the 2 years are analyzed and discussed further in this article.

Limoges et al. (2022) conducted a field study comparing the survival of *E. coli* in northeastern US soils amended with composted and heat‐treated BSAAO. The study reported that *E. coli* survival remained higher in soils amended with HTPP and PL than in unamended plots. Similar results of higher persistence of *E. coli* TVS353 have been observed in previous studies conducted under different experimental conditions (Sharma and Reynnells 2016; Litt et al. 2021). As shown in Table S1, the nutrient composition of PL and HTPPs varied between years, with higher levels of total nitrogen (N) and phosphorus (P) observed in Year 1. The extended survival of *E. coli* in these amendments is often attributed to nutrient availability, which has been previously identified as a strong driver of *E. coli* survival in soils (Franz et al. 2008; Sharma et al. 2019). However, post‐amendment soil samples were not collected to analyze the nutrient content over time. Future studies should also focus on monitoring the temporal changes in soil nutrient availability, pH, and organic matter content to better understand their relationship with *E. coli* survival dynamics. Additionally, the reduction of competing microbial species in HTPP due to the pelleting process may contribute to greater survival of *E. coli* in HTPP than in PL‐amended soils (Chen and Jiang 2017; Kharel et al. 2025). In contrast, no significant difference in survival was found during Year 2. In previous studies that have reported higher survival in BSAAOs, soils were sampled from fields in the Northeastern and Mid‐Atlantic US, which have climate and soil properties different from those of Georgia (Polsky et al. 2000; Sheppard et al. 2002). These regions generally experience cooler temperatures, higher annual precipitation, and different soil types, whereas soils in Georgia may be prone to faster drying and desiccation stress. Further, the type of plant growth influences the survival of bacterial populations both on the produce and in the soil in which the produce is grown (Islam et al. 2004). Shah et al. (2019) studied the survival of *S. enterica* in HTPP‐amended plots with or without spinach plants. Although spinach is not a root crop, the results from their study also showed no significant differences in supporting survival. Phenolic compounds such as flavonoids and alkenyl cysteine sulfoxides (ACSOs) contained in onions have antimicrobial properties, bactericidal to *Salmonella* Typhimurium and *E. coli* (Johnson and Vaughn 1969; Crozier et al. 1997; Hollman and Arts 2000; Islam et al. 2004; Brglez Mojzer et al. 2006). However, phenolic compounds or root exudates released by onions during field growth were not within the scope of the study. Future research is required to determine the composition of phenolic compounds released by onions to better understand their impact on *E. coli* survival.

The decline in bacterial populations was not consistent across soil treatment plots (Figures 4, 5, 6, 7 ). Over both years, there were instances of increases in *E. coli* population levels and sustained persistence across consecutive soil samplings. These fluctuations in bacterial populations are common in field experiments due to varying environmental conditions (Vidovic et al. 2007; Pang et al. 2017, 2018, 2020). Limoges et al. (2022) observed a slight increase in the *E. coli* population at 3 DPI, followed by a decline over 104 and 102 DPI, respectively, with some fluctuations in bacterial concentration throughout the sampling days in composted and heat‐treated poultry amendments. In contrast, survival studies based on laboratory and greenhouse experiments performed under uniformly controlled experimental conditions do not show fluctuations in declining bacterial populations (Baker et al. 2021; Bardsley et al. 2022; Gutierrez and Schneider 2022). Çekiç et al. (2017) observed differences in the survival of *E. coli* in raw bovine manure‐amended soil under laboratory and field conditions. However, the authors observed an increase in the *E. coli* population on the first day of postinoculation sampling, likely due to the acclimatization of manure‐borne *E. coli*. These differences in survival trends under laboratory/greenhouse and field conditions can be attributed to the dynamic environmental conditions. Previous research has shown variability in the prevalence of *Salmonella* and *Listeria*, even within the same growing region and across different farms, due to factors such as soil moisture fluctuations, pH differences, precipitation patterns, farm management practices, and proximity to animal operations (Strawn et al. 2013, Strawn et al. 2014). Log‐linear, Geerard, Weibull, or biphasic survival models have been fitted to most of the survival data from laboratory‐based and controlled greenhouse studies (Franz et al. 2008; Ongeng et al. 2011, 2014). However, these are not suitable for modeling microbial survival in agricultural field environments due to variable decline. Hence, the LME model in the present study enabled us to incorporate various weather factors that help explain *E. coli* survival in agricultural environments. Microbial populations often respond to fluctuating weather conditions, including heavy rain, dry periods, and temperature extremes, with a lag (Mellefont et al. 2004; Nev et al. 2021). To account for that, this study further modeled the lagged environmental variables on the survival of *E. coli* across different biological soil amendments and HTPP plots with and without onions (Figure 7).

**FIGURE 4:**
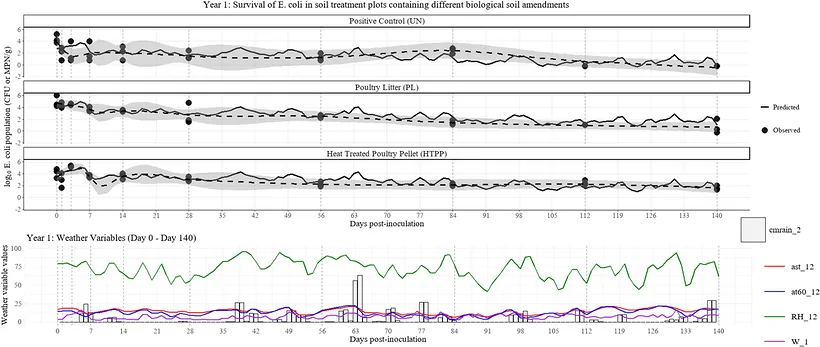
“Year 1” fitted linear‐mixed‐effect (LME) model for the survival of *Escherichia coli* in soil treatment plots containing different biological soil amendments from Days 0 to 140. The first three graphs display the survival of *E. coli* in unamended positive control (UN), poultry litter (PL), and heat‐treated poultry pellets (HTPP). The last graph illustrates the significant weather factors. The observed values of the *E. coli* population are depicted using ●, dashed vertical lines indicate sampling days (0, 1, 3, 7, 14, 28, 56, 84, 112, 140, 161), and —— depicts the predicted line. A black dashed line (——) represents a smoothed trend for observed counts, included solely for visualization purposes to help identify overall trends, with the shaded region indicating the variation among replicates. The cmrain_2_ is the cumulative rainfall 2 days before sampling; RH12 is the relative humidity averaged over the 2 days before sampling; at6012 is the air temperature averaged over the 2 days before sampling; ast12 is the soil temperature averaged over the 2 days before sampling; and W1 is the wind speed 1 day before sampling. MPN, Most Probable Number.

**FIGURE 5:**
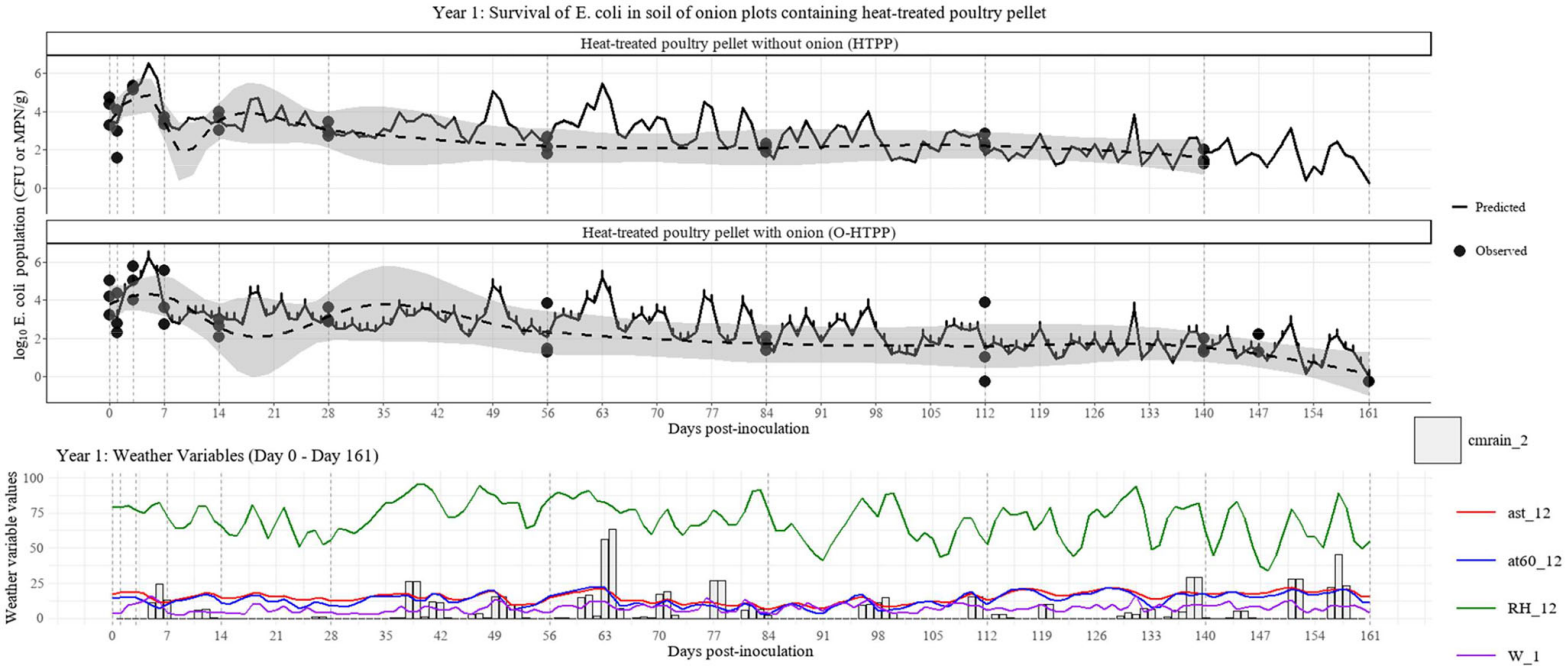
“Year 1” fitted linear‐mixed‐effect (LME) model for the survival of *Escherichia coli* in the soil of onion plots containing heat‐treated poultry pellet from Days 0 to 161. The first two graphs illustrate the survival of *E. coli* in heat‐treated poultry pellets with and without onions (HTPP, O‐HTPP), whereas the last graph displays the significant weather factors. The observed values of the *E. coli* population are depicted using ●, dashed vertical lines indicate sampling days (0, 1, 3, 7, 14, 28, 56, 84, 112, 140, 161), and —— depicts the predicted line. A black dashed line (— —) represents a smoothed trend for observed counts, included solely for visualization purposes to help identify overall trends, with the shaded region indicating the variation among replicates. The cmrain_2_ is the cumulative rainfall 2 days before sampling; RH12 is the relative humidity averaged over the 2 days before sampling; at6012 is the air temperature averaged over the 2 days before sampling; ast12 is the soil temperature averaged over the 2 days before sampling; and W_1_ is the wind speed 1 day before sampling. MPN, Most Probable Number.

**FIGURE 6:**
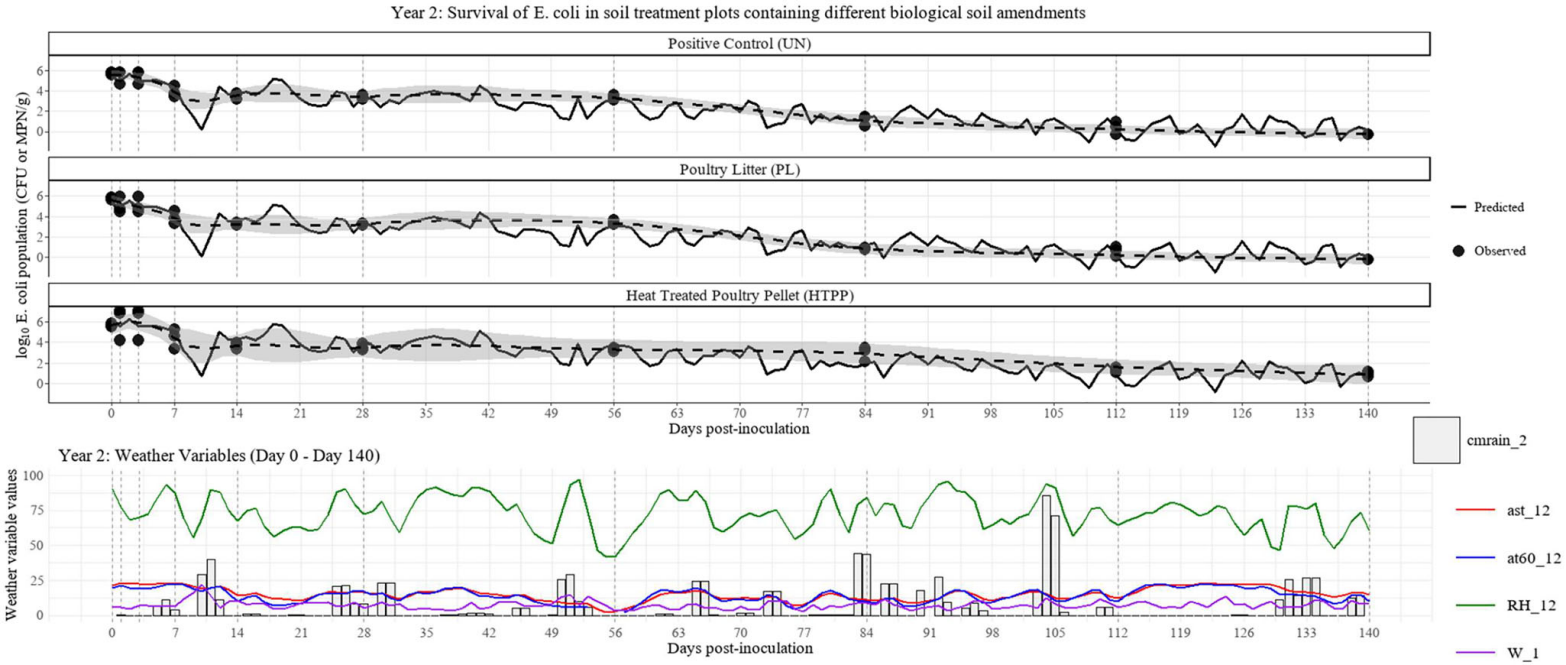
“Year 2” fitted linear‐mixed‐effect (LME) model for the survival of *Escherichia coli* in soil treatment plots containing different biological soil amendments from Days 0 to 140. The first three graphs display the survival of *E. coli* in unamended positive control (UN), poultry litter (PL), and heat‐treated poultry pellets (HTPP). The last graph illustrates the significant weather factors. The observed values of the *E. coli* population are depicted using ●, dashed vertical lines indicate sampling days (0, 1, 3, 7, 14, 28, 56, 84, 112, 140, 161), and —— depicts the predicted line. A black dashed line (— —) represents a smoothed trend for observed counts, included solely for visualization purposes to help identify overall trends, with the shaded region indicating the variation among replicates. The cmrain_2_ is the cumulative rainfall 2 days before sampling; RH12 is the relative humidity averaged over the 2 days before sampling; at6012 is the air temperature averaged over the 2 days before sampling; ast12 is the soil temperature averaged over the 2 days before sampling; and W1 is the wind speed 1 day before sampling. MPN, Most Probable Number.

**FIGURE 7:**
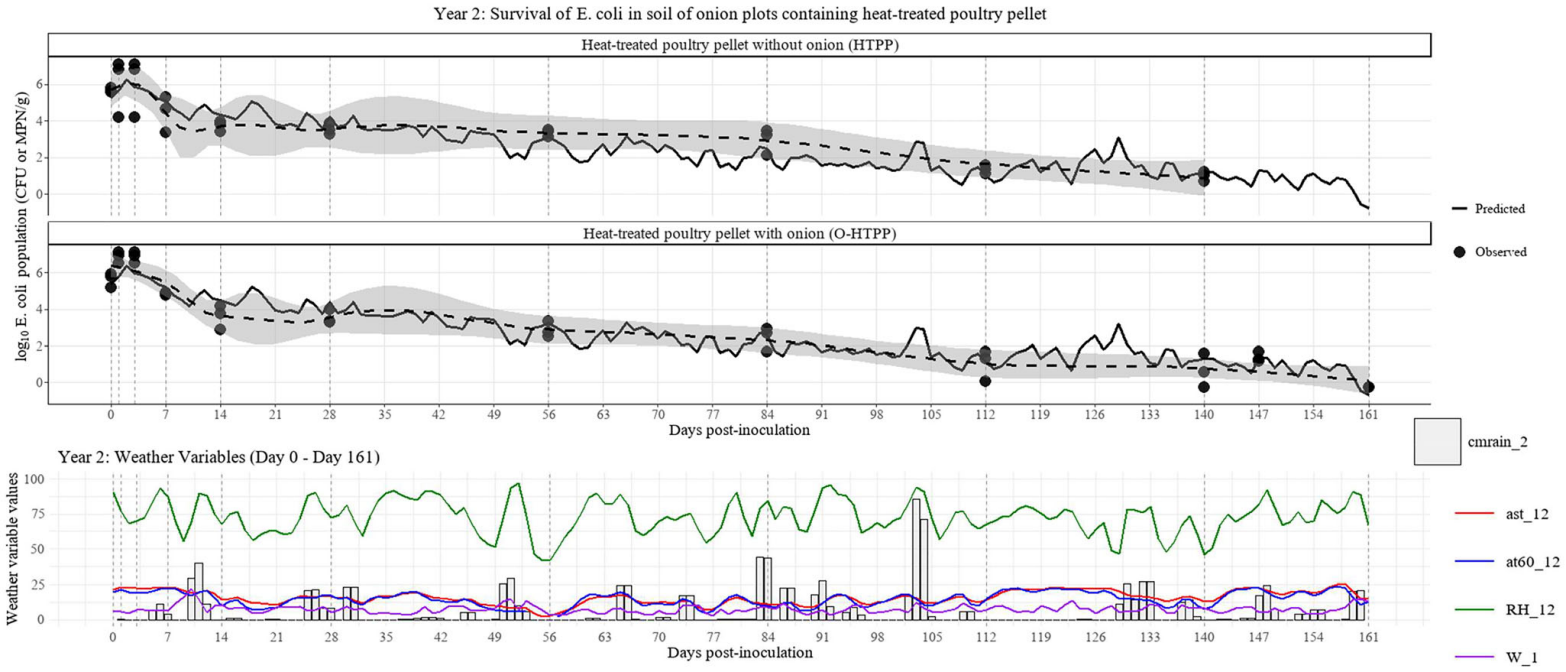
“Year 2” fitted linear‐mixed‐effect (LME) model for the survival of *Escherichia coli* in the soil of onion plots containing heat‐treated poultry pellet from Days 0 to 161. The first two graphs illustrate the survival of *E. coli* in heat‐treated poultry pellets with and without onions (HTPP, O‐HTPP), whereas the last graph displays the significant weather factors. The observed values of the *E. coli* population are depicted using ●, dashed vertical lines indicate sampling days (0, 1, 3, 7, 14, 28, 56, 84, 112, 140, 161), and —— depicts the predicted line. A black dashed line (— —) represents a smoothed trend for observed counts, included solely for visualization purposes to help identify overall trends, with the shaded region indicating the variation among replicates. The cmrain_2_ is the cumulative rainfall 2 days before sampling; RH12 is the relative humidity averaged over the 2 days before sampling; at6012 is the air temperature averaged over the 2 days before sampling; ast12 is the soil temperature averaged over the 2 days before sampling; and W1 is the wind speed 1 day before sampling. MPN, Most Probable Number.

During Year 1, recovery of *E. coli* populations (0.2–2.4 log_10_ CFU or MPN/g) was observed on certain sampling days in the soil‐only plots (Figure 4). Relative humidity was higher on the days when it rained throughout the study (Figure 2). Rainfall events ranged from a minimum of 0.24 mm to a maximum of 56 mm. Accordingly, the average relative humidity on those days was around 83%, compared with 64% on the other days. An increase in relative humidity due to rainfall events has been reported to support the survival and growth of *E. coli* in soil (Ishii et al. 2006; Ishii et al. 2010). Although the weather variable associated with rainfall was not statistically significant in Year 1, its indirect effect on RH may have contributed to *E. coli* survival. It also indicates moisture availability as a critical factor in supporting *E. coli* survival. This finding is also consistent with studies that have reported high relative humidity associated with rainfall events positively influencing the survival of *E. coli* in manure‐amended soils (Pang et al. 2018, 2020). Although more days with low RH extremes were observed in Year 2, they occurred later in the season, when *E. coli* populations already tend to decline due to natural die‐off. Here, an increase in the *E. coli* population might indicate the resuscitation of viable bacterial cells that were previously nonculturable and therefore remained undetected (Roszak and Colwell 1987). Shah et al. (2019) studied the roles of RpoS and the formation of viable but nonculturable (VBNC) cells in the survival of *S. enterica* in HTPP‐amended and unamended soils. The authors reported similar levels of recovery for wild‐type and *RpoS*‐deficient strains using qPCR and plate count methods, indicating that all bacterial cells were viable and culturable in HTPP‐amended soils due to sufficient moisture from irrigation, which resulted in high water‐extractable carbon and nitrogen contents.

Wind can negatively affect the survival of bacterial populations in the soil through several mechanisms, including increased UV exposure, soil desiccation due to moisture loss by evaporation, and surface disturbance of bare soil (Ravva et al. 2011; Qiu et al. 2022). Pang et al. (2018) reported that a wind speed greater than 4 km/h 2 days before sampling may lower the prevalence and levels of *E. coli* in mixed produce and dairy farms. In the present study, wind speed was a significant negative factor during Year 2, fluctuating from a minimum of 2.7 km/h to a maximum of 21.8 km/h (Figure 2). According to the Beaufort wind scale, the average wind speed falls under a gentle breeze (National Weather Service 2025), which suggests that the desiccation effect of high‐speed winds may reduce bacterial survival by reducing surface moisture. In contrast, Year 1 experienced fewer high‐wind events and Year 2 experienced more frequent and earlier extreme wind events (*z* > 2), which cumulatively increased throughout the study (Figure 3a). Unlike in the second year of the study, where wind speed had a negative effect, the model identified a positive impact of wind speed on onion yields in the HTPP plots during Year 1. These opposing effects likely reflect differences in the timing, frequency, and magnitude of extreme wind‐speed events, as well as interactions with onions growing in the plot. Moreover, cmrain_2_ positively influenced survival during Year 2, likely interacting with high‐wind‐speed events to counter the desiccation effect. In contrast, Year 1 exhibited fewer high‐wind‐speed events, which occurred much later in the season, when the onion plants may have already developed and provided protective cover to the soil surface. The positive effect of wind speed observed in Year 1 could be attributed to enhanced surface aeration, which may benefit *E. coli* in the microenvironments created by onions growing in the plots. However, further research is needed to investigate the interactive effects of wind speed and vegetation cover under different crop growth stages to better understand how canopy development modulates wind effects on soil microclimate and *E. coli* survival.

Furthermore, in Year 2, soil temperature positively affected *E. coli* survival across treatment plots, with or without sweet onions. During the second year of the study, the average soil temperature was 16°C, with a daily range of 2–26°C (Figure 2). In comparison, previous microcosm laboratory studies have reported the prolonged persistence of enteric bacteria in soil at cooler temperatures compared to warmer ones (Ishii et al. 2006; Çekiç et al. 2017). Underthun et al. (2018) stated that soil temperature is an influential variable on bacterial decay and reported higher persistence and recovery of *E. coli* at later sampling days from soil microcosms incubated at 20°C compared to those incubated at 30°C. Continuous incubation of the soil microcosm at 30°C in laboratory settings may accelerate die‐off due to heat stress. Although field temperatures were moderate and variable, Year 2 exhibited lower temperature extremes. Similarly, temperature and temporal data reported in another study showed that *E. coli* populations survived well in warmer temperatures (McKenzie‐Reynolds et al. 2025). Additionally, air temperature negatively affected *E. coli* survival across the soil treatment plots, with or without sweet onions, in Year 2. It alternated between −5°C and 24°C and averaged at 15°C (Figure 2). Tran et al. (2020) conducted a meta‐analysis summarizing the quantitative effects of several environmental drivers (e.g., temperature, moisture, and solar radiation) on the rate of decline of *E. coli* in manure‐amended soils. The air temperature was identified as the most prominent factor affecting the persistence of *E. coli* under field conditions. Another study reported a slow decline in *E. coli* during seasons with lower ambient temperatures (8–11°C) due to reduced UV‐B radiation (Sharma et al. 2019). Soil and air temperatures had opposite effects on the survival of *E. coli*. In general, soil temperature reflects ambient temperature and directly affects *E. coli* survival. However, air temperature may indirectly affect the soil environment, influencing evaporation rates and moisture levels. Moreover, significant wind speeds during Year 2 may have enhanced the cooling effect on air temperature and contributed to desiccation, whereas soil temperature acts as a buffer against dynamic environmental stresses. In contrast, Year 1 exhibited stable thermal conditions, with only 1 day of extreme lows in air and soil temperatures. Similar positive and negative effects of soil and air temperatures on *E. coli* survival were observed in HTPP‐managed plots with and without onions. The onion leaves may have provided partial shading, reducing surface heating and slightly buffering soil temperatures against rapid changes in air temperature. The negative association between air temperature and *E. coli* survival suggests that the upper soil layers remained vulnerable to thermal and evaporative stress, particularly under higher daytime temperatures and a significant wind‐speed effect.

Cumulative rainfall over 2 days positively influenced *E. coli* survival in the HTPP‐managed sweet onion plots. Litt et al. (2021) reported that higher cumulative rainfall in each 10‐day interval significantly increased the survival duration of *E. coli* in soils amended with composted and HTPPs. Several studies have found that increases in soil moisture from rainfall events significantly affect the survival of *E. coli* in agricultural soils (Chandler and Craven 1980; Underthun et al. 2018; Pang et al. 2020). During Year 2, extreme rainfall events occurred earlier and more frequently, with cumulative counts increasing progressively throughout the study period. Rainfall prevents desiccation and facilitates the diffusion of nutrients and substrates, enhancing their availability to bacterial populations (Chandler and Craven 1980; Zak et al. 1999; Vidovic et al. 2007). Additionally, root zone moisture retention may have been enhanced by plant transpiration cycles, supporting microbial viability during warmer days, as soil temperature was also significant in Year 2.

This longitudinal study represents one of the first modeling approaches to quantify the effects of field environmental conditions on *E. coli* survival in PL‐based amended soils. Separate LME models were developed for each growing season to account for year‐specific variability in environmental extremes. Modeling years independently allowed us to better capture the temporal dynamics of *E. coli* survival and to quantify the direction and magnitude of weather effects under distinct seasonal contexts. Despite the temporal differences, several consistent patterns emerged across years. Soil temperature, rainfall, and relative humidity were positively associated with *E. coli* survival, whereas higher wind speed and elevated air temperature were negatively associated with *E. coli* survival. However, adding more data to the model through more frequent sampling will further improve predictions. Further incorporating bacterial decay rates and additional weather‐variable lags may also help enhance model accuracy in predicting surviving bacterial populations.

Furthermore, as onions are root vegetables, with the harvestable portion of the crop directly in contact with the soil, they are exposed to bacteria in BSAAO and soils (Islam et al. 2005). Literature has also reported the risk of contamination of field‐grown produce when BSAAOs are used. A similar study was recently published that examined the transfer of *E. coli* from manure‐amended soils to cucumbers growing in the field (Litt et al. 2021). In that study, *E. coli* was recovered from approximately 97.5% (156/160) and 35% (63/180) of cucumbers grown in soils amended with BSAAO. Islam et al. (2004) detected *E. coli* O157:H7 on onions and carrots grown in fields contaminated with manure. Similarly, McKenzie‐Reynolds et al. (2025) detected *E. coli* contamination in cucumber, cantaloupes, radish, and spinach grown in PL (6‐month‐old) and HTPP plots at 90 DPI. However, the potential for contamination and the occurrence of *E. coli* declined by 120 DPI, with only two spinach plants detected as positive. Onion bulbs are usually harvested from the field and either cured with forced air in regions with high precipitation or left on the ground after harvest in drier climates (Suslow 2012). In the present study, onions were lifted from the soil and left on the ground to cure for 2 weeks. The prevalence of *E. coli* decreased, as did its levels in the O‐HTPP plots. Moyne et al. (2022) recovered *E. coli* in 19 of 25 onion bulbs (95%) lifted 7 days postinoculation. Similarly, Emch and Waite‐Cusic (2016) reported a decrease in prevalence from 13.3% (20 out of 150) to 4% (6 out of 150) after curing. Hence, onion curing practices appear to reduce foodborne pathogens due to several factors, including the desiccation of onion bulbs through UV exposure, the antimicrobial properties of onion skin extracts, and low nutrient availability (Takahama and Hirota 2000; Emch and Waite‐Cusic 2016).

## 5 Conclusion

In conclusion, this 2‐year field study quantified the effects of poultry‐litter‐based amendments and field environmental conditions on the survival of *E. coli* in Georgia soils and sweet onion cropping systems. Survival was higher in soils amended with HTPPs and composted PL compared to unamended soils, particularly in Year 1. LME models identified key weather parameters, including relative humidity, air and soil temperature, rainfall, and wind speed, as significant predictors of *E. coli* survival, with their influence varying between years. The presence of onions in HTPP‐managed plots did not significantly affect *E. coli* survival in soils, and field curing resulted in a substantial decline in *E. coli* levels on the onion surface. Findings from the study underscore that moderate variability in weather conditions may not be sufficient to explain *E. coli* survival. Instead, differences in extreme weather events that were significant predictors between Years 1 and 2 indicated an association with the survival outcomes. These results suggest that future modeling efforts should account for pathogen decay patterns, intensity, and frequency of extreme weather events during the production season to improve the prediction accuracy under dynamic field conditions. Findings from this study advance our scientific understanding of how amendment type and extreme environmental variability shape the survival dynamics of enteric bacteria in agricultural soils. Future research should also evaluate *E. coli* survival across diverse geographic locations with varying environmental conditions and topographical characteristics to inform site‐adapted risk‐mitigation strategies. Comparative studies assessing survival under organic field management practices are also needed to inform risk‐based recommendations for organic growers.

## Conflicts of Interest

The authors declare no conflicts of interest.

## Supporting information




**Supporting Table S1**. “Year 1” chemical analysis of heat‐treated poultry pellet (HTPP) and composted poultry litter (PL).
**Supporting Table S2**. “Year 2” chemical analysis of heat‐treated poultry pellet (HTPP) and composted poultry litter (PL).

## Data Availability

The data that support the findings of this study are available on request from the corresponding author. The data are not publicly available due to privacy or ethical restrictions.
