# Supplementary material for: Temporal and Environmental Influences on the Survival of Escherichia coli in Poultry Litter‐Based Soil Amendments in Georgia Sweet Onion Production Systems
Source: Risk Anal. 2026 Sep 30;46(10):e70330. doi: 10.1111/risa.70330 (PMC13625810; doi:10.1111/risa.70330)
Supplement: Supplementary file 1 [file RISA-46-0-s001.docx]

**Supplementary Material**

Table S1‘Year 1’ Chemical analysis of heat-treated poultry pellet (HTPP) and composted poultry litter (PL)

| **Soil Amendment** | **Total solids (mg/L)** | **Total Ash (mg/L)** | **K (mg/L)** | **P (mg/L)** | **Mn (mg/L)** | **Cu (mg/L)** | **Zn (mg/L)** | **NH_3_ (mg/L)** | **Total Kjeldahl nitrogen (mg/kg)** | **Moisture (%)** | **pH** |
| --- | --- | --- | --- | --- | --- | --- | --- | --- | --- | --- | --- |
| HTPP | 814,942 | 31,897 | 41,548 | 14,898 | 483.84 | 608.3 | 470.48 | 1,828 | 22,047 | 18.5 | 7.1 |
| PL | 693,870 | 495,441 | 13,762 | 5,013 | 218.52 | 303.45 | 203.5 | 1,379 | 2,485 | 30.6 | 8.5 |

Table S2 ‘Year 2’ Chemical analysis of heat-treated poultry pellet (HTPP) and composted poultry litter (PL)

| **Soil Amendment** | **Ca (mg/kg)** | **Mg**  **(mg/kg)** | **S (mg/kg)** | **K (mg/kg)** | **P (mg/kg)** | **Mn (mg/)** | **Cu (mg/l)** | **Zn (mg/l)** | **Fe (mg/l)** | **Al (mg/l)** | **B**  **(mg/l)** | **Na (mg/l)** | **Total nitrogen (mg/kg)** |
| --- | --- | --- | --- | --- | --- | --- | --- | --- | --- | --- | --- | --- | --- |
| HTPP | 2,610 | 395 | 485 | 2,410 | 1,850 | 485 | 597 | 495 | 1,521 | 1,729 | 115 | 10,049 | 34,450 |
| PL | 8,411 | 2,733 | 2,256 | 13,403 | 13,355 | 172 | 145 | 139 | 689 | 1,244 | 18.2 | 1,557 | 11,220 |
